# Localized mammographic density is associated with interval cancer and large breast cancer: a nested case-control study

**DOI:** 10.1186/s13058-019-1099-y

**Published:** 2019-01-22

**Authors:** Fredrik Strand, Edward Azavedo, Roxanna Hellgren, Keith Humphreys, Mikael Eriksson, John Shepherd, Per Hall, Kamila Czene

**Affiliations:** 1 Department of Medical Epidemiology and Biostatistics, Karolinska Institutet, Nobels Väg 12A, 171 77 Stockholm, Sweden; 2 Breast Radiology, Karolinska University Hospital, Stockholm, Sweden; 3 Department of Molecular Medicine and Surgery, Karolinska Institutet, Stockholm, Sweden; 4 Department of Radiology, Southern General Hospital, Stockholm, Sweden; 5 University of Hawaii Cancer Center, Honolulu, USA; 6 Department of Oncology, South General Hospital, Stockholm, Sweden

**Keywords:** Breast cancer, Mammography, Screening, Mammographic density, Early detection

## Abstract

**Background:**

High mammographic density is associated with breast cancer and with delayed detection. We have examined whether localized density, at the site of the subsequent cancer, is independently associated with being diagnosed with a large-sized or interval breast cancer.

**Methods:**

Within a prospective cohort of 63,130 women, we examined 891 women who were diagnosed with incident breast cancer. For 386 women, retrospective localized density assessment was possible. The main outcomes were interval cancer vs. screen-detected cancer and large (> 2 cm) vs. small cancer. In negative screening mammograms, overall and localized density were classified reflecting the BI-RADS standard. Density concordance probabilities were estimated through multinomial regression. The associations between localized density and the two outcomes were modeled through logistic regression, adjusted for overall density, age, body mass index, and other characteristics.

**Results:**

The probabilities of concordant localized density were 0.35, 0.60, 0.38, and 0.32 for overall categories “A,” “B,” “C,” and “D.” Overall density was associated with large cancer, comparing density category D to A with OR 4.6 (95%CI 1.8–11.6) and with interval cancer OR 31.5 (95%CI 10.9–92) among all women. Localized density was associated with large cancer at diagnosis with OR 11.8 (95%CI 2.7–51.8) among all women and associated with first-year interval cancer with OR 6.4 (0.7 to 58.7) with a significant linear trend *p* = 0.027.

**Conclusions:**

Overall density often misrepresents localized density at the site where cancer subsequently arises. High localized density is associated with interval cancer and with large cancer. Our findings support the continued effort to develop and examine computer-based measures of localized density for use in personalized breast cancer screening.

**Electronic supplementary material:**

The online version of this article (10.1186/s13058-019-1099-y) contains supplementary material, which is available to authorized users.

## Background

High mammographic density is associated with an increased absolute risk of breast cancer [1], with interval cancer compared to screen-detected cancer [2–5], and with large cancer compared to small cancer [6]. The proportion of bright and dark areas in the mammogram forms the basis for density classification, either by the visual Breast Imaging Reporting and Data System (BI-RADS) categorization or by automated computer-based approaches [7, 8]. Current proposals to individualize breast cancer screening are often based on assessments of overall density [9]. Screening has successfully decreased breast cancer mortality through early mammographic detection [10, 11]. The outcomes of interval cancers and large cancers represent failures of early detection. Interval cancer is a cancer that emerges after a negative screening and is the result either of mammographic masking by overlapping tissue or of fast tumor growth [12–14]. Both interval cancer and large-sized cancer worsen patient survival [15–17].

Moving beyond overall density, it has been shown that breast cancer is more likely to arise in localized areas of high density [18, 19]. For two women with the same overall mammographic density, the cancer in one woman might develop in a localized high-density area while the cancer in the other woman might develop in a localized low-density area. Histopathological studies have shown that the local tumor environment is of importance for cancer initiation and progression [20, 21]. One measure of the local tissue composition is the mammographic density in the prior negative mammogram at the site of the subsequent cancer. In addition to potentially influencing the tumor initiation and growth, localized high density could also serve to mask a cancer. Therefore, the latest edition of the BI-RADS lexicon emphasizes that when visually categorizing the overall mammographic density one should take into account the existence of localized areas of high density that could potentially mask cancer [7]. Thus, localized density assessment is emerging as a potentially important consideration in designing screening programs. However, there is a lack of studies directly examining how increased localized density impacts screening outcomes.

The current study was conducted within a prospective cohort of more than 63,130 women who regularly participated in screening mammography. We determined the concordance probability between overall density and localized density at the site of the subsequent cancer. We examined whether localized density, at the site of the subsequent cancer, is independently associated with being diagnosed with a large-sized or interval breast cancer—both of which are related to delayed detection.

## Patients and methods

The study population was based on incident breast cancers in the Swedish KARolinska MAmmography Project for Risk Prediction of Breast Cancer (KARMA) cohort [22]. In summary, KARMA is a prospective study cohort for which inclusion started in January 2011 and includes 70,871 women attending mammography screening or clinical mammography at four hospitals in Sweden. Upon study entry, participants donated blood and filled out a detailed web-based questionnaire. Participants were asked to report on the reproductive history, use of oral contraceptives and hormone replacement therapy, previous benign breast disease, and family history of breast cancer. Body mass index (BMI) was calculated based on self-reported height and weight. All participants provided written informed consent, and the study was approved by the ethical review board at Karolinska Institutet.

For the present study, we included all women in the KARMA cohort who had available mammograms and were not diagnosed with breast cancer before entering the cohort. The overall study population (*n* = 63,130) contained both women with incident breast cancer (*n* = 891) and healthy controls (*n* = 62,239). Localized density assessments were obtainable only for a limited subset of women with incident breast cancer (*n* = 386) since for the other women the prior screening mammograms were not available (*n* = 471) or the cancer was occult (*n* = 34) and not localizable. The derivation of the study populations has been specified in detail in Additional file 1: Figure S1. Data on cancer diagnoses including cancer characteristics were obtained through linkage with the Swedish Cancer Register and the Breast Cancer Quality Register at the Regional Cancer Centre Stockholm-Gotland using the Swedish personal identity numbers.

Mammographic density was assessed on a percent scale using the validated fully automated STRATUS software [23]. It was then categorized by cut-points (2%, 18%, 49%) into four groups (cBIRADS) reflecting the definitions by the American College of Radiology Breast Imaging Reporting and Data System (BI-RADS) lexicon, fifth edition [7]. The categories are as follows: “A” (the breasts are almost entirely fatty), “B” (there are scattered areas of fibroglandular density), “C” (the breasts are heterogeneously dense, which may obscure small masses), and “D” (the breasts are extremely dense, which lowers the sensitivity of mammography). Localized density was visually categorized according to these BI-RADS criteria applied to the localized area instead of the whole breast. Assessments were based on consensus decision between two radiologists blinded to the detection mode of the cancer, i.e., whether it was screen-detected or an interval cancer. First, the ipsilateral mediolateral oblique view mammogram from time of diagnosis was reviewed to identify the location of the cancer. Second, the radiologists reviewed the corresponding location in the ipsilateral prior negative screening mammogram and assessed the localized density within an approximately 3 by 3 cm area. In addition, any prior tumor signs were assessed according to the interval cancer classification outlined in the European Union guidelines for quality assurance in breast cancer screening [24]. The potential tumor appearance in the prior negative mammogram was classified into true, minimal sign, or false negative.

### Methods—statistical analysis

The main outcomes for our analyses were interval cancer and large cancer (> 2 cm). The cut-point for large cancer was chosen to reflect the T2 stage in the TNM system [25]. Cancer size measures were based on post-operative specimen pathology assessment. Interval cancer was defined as cancer that was clinically diagnosed within 2 years of a negative screening mammogram. Two years is the normal mammography screening interval in Sweden. Women with occult breast cancer, where it was not possible to determine the tumor location at the time of diagnosis, were not included in the study.

The Pearson’s correlation coefficient between overall and localized density was calculated. We fitted a multinomial regression model to estimate probabilities of having a certain localized density category for each overall density category. Logistic regression models were fitted with overall density as the predictor for each of the four different outcomes: interval cancer compared to healthy, interval cancer compared to screen-detected cancer, large cancer compared to healthy, and large cancer compared to small cancer. Odds ratios (OR) were estimated crude, age- and BMI-adjusted, and multiple adjusted for the following potential confounders: age, BMI, menopausal status, age at menarche, alcohol use, parity, use of hormone replacement therapy, ever use of oral contraceptives, benign breast disease, and family history of breast cancer. Missing data in any adjustment variable was handled by creating a missing category. In the analysis of data from individuals with localized density information, localized density was used as the predictor of the two outcomes: interval cancer compared to screen-detected cancer and large cancer compared to small cancer. The models using localized density as a predictor were additionally adjusted for overall density. Our final analysis focused on interval cancers arising within 12 months after a negative screening mammogram with the intent to focus on cancers that were more likely masked—in line with a prior study examining potential masking measures [26].

All statistical tests were two-sided with pre-determined cutoff for statistical significance at alpha = 0.05. The computer software Stata, version 14, was used for all statistical analyses.

## Results

Patient and cancer characteristics are described in Table 1. Overall, there were 891 women diagnosed with incident breast cancer, and localized density assessment was possible for a subset of 386 women. Among all characteristics, only BMI differed between the overall and the subset population: 25.7 kg/m^2^ in the overall and 26.0 kg/m^2^ in the localized density-assessed subset (*p* = 0.043). In the overall population, there were 246 women with interval cancer and 250 women with large cancer. Of the women with large cancer, 168 were screen-detected and 82 were interval cancers. The median time between the preceding screening examination and the diagnostic examination was 1.99 years.

**Table 1:**
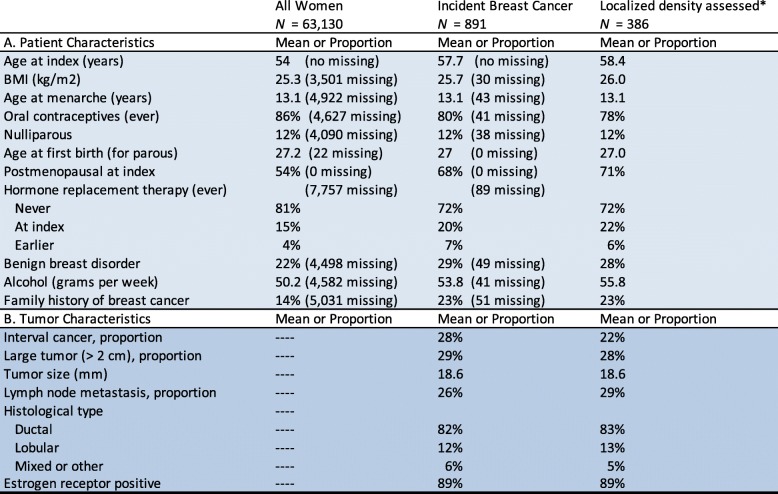
Study population and tumor characteristics, including test of difference between women with cancer compared to healthy, as well as between women with localized density assessments and without such assessment

*Localized density assessed: image availability and tumor appearance allowing local density assessment for women with breast cancer

**Fig. 1:**
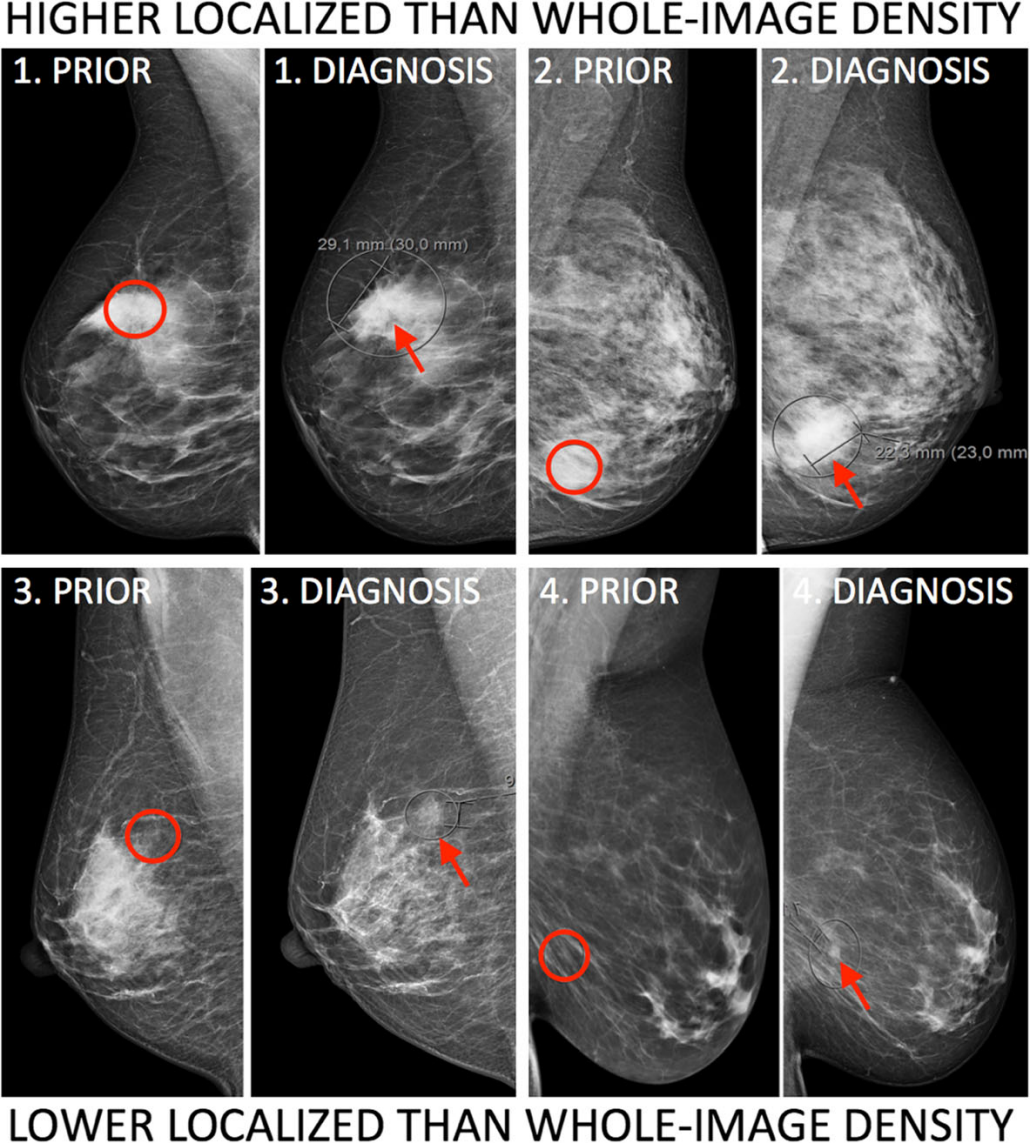
Examples of cases with discordant overall and localized density. DIAGNOSIS denotes the mammogram at the time of diagnosis. PRIOR denotes the mammogram from the prior screening (median time 1.99 years before DIAGNOSIS). DIAGNOSIS was used to localize the tumor (red arrow), while PRIOR was used to assess the overall and localized (red circle) density

**Fig. 2:**
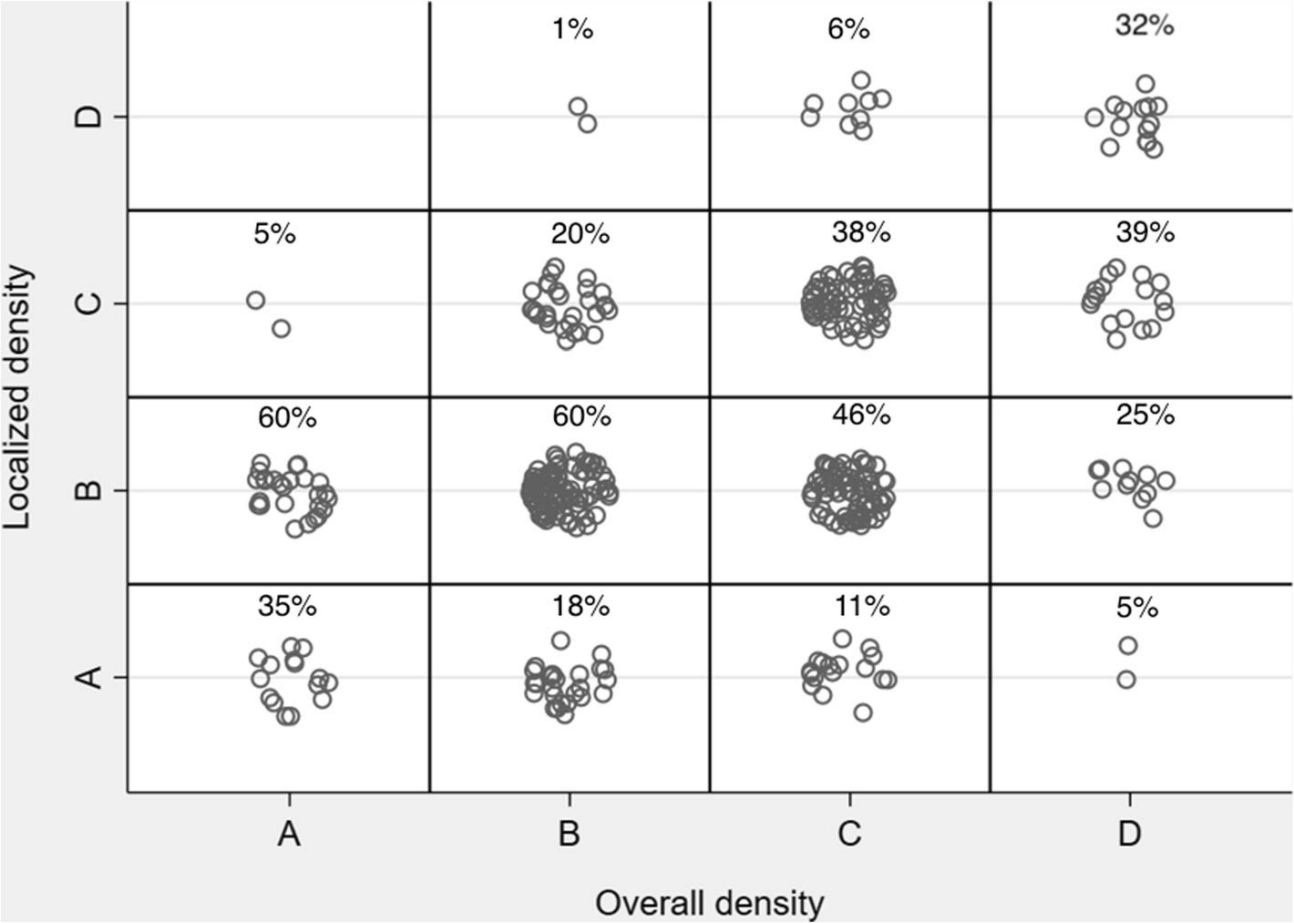
Distribution across categories of the overall and localized mammographic density of the 386 women with incident breast cancer for which local density assessment was performed. Category A is the least dense, and category D is the most dense. Marker placements within each rectangle carry no meaning; jittered for enhanced visibility. There is a moderate correlation between the two density parameters (*r* = 0.42). Percentage numbers show the proportion of cases within each overall density category

Figure 1 shows examples of mammograms with discordant overall and localized density categories, while Fig. 2 shows a plot of overall and localized density categories for all assessed women. The Pearson’s correlation coefficient between overall and localized density was *r* = 0.42. For women with overall density: 21% of the women with overall density B had a higher category local density. Fifty-seven percent of the women with overall density C had a lower category local density.

In Additional file 2: Table S1, we describe the tumor size for each pair of concordant-discordant overall and localized density. For overall density A, the average tumor size at concordance was 10.5 mm compared to 16.5 mm for discordant localized category B. For overall density category B, the average tumor size was 18.4 mm at concordance and 12.6 mm at discordant localized category A. Based on these descriptive tumor size measures, it seemed that making tumor size predictions only based on the overall density category would be far from adequate considering the large size difference related to localized density.

Table 2 reports the estimated odds ratios of interval cancer and large cancer for the full study population (*n* = 63,130). For women with overall density category D compared to category A, the OR for interval cancer vs. remaining healthy and interval cancer vs. screen-detected cancer were 31.5 (95%CI 10.8 to 91.8) and 12.7 (95%CI 3.9 to 40.7) respectively, after multiple adjustments. The OR for large cancer vs. remaining healthy and large cancer vs. small cancer were 14.9 (95%CI 6.7 to 33.1) and 4.6 (95%CI 1.8 to 11.6) respectively, after multiple adjustments. For each outcome, the linear trend was significant.

**Table 2:**
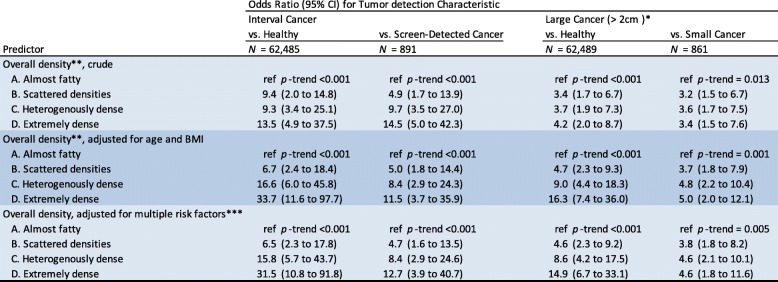
Overall density as predictor of interval cancer and large cancer. Odds ratios estimated by logistic regression modeling

*Tumor size was based on post-operative specimen pathology assessment; missing for 8 cases

**Overall density was categorized on percent density scale cut-points (2%, 18%, 49%) into four groups reflecting the definitions by the American College of Radiology Breast Imaging Reporting and Data System (BI-RADS) lexicon, fifth edition

***Adjusted for age, menopausal status, body mass index, age at menarche, alcohol use, parity, use of hormone replacement therapy, use of oral contraceptives, family history, and benign breast disease

Table 3 shows the estimated odds ratios of interval cancer and large cancer for the localized density-assessed subset of women (*n* = 386). For women with localized density category D compared to category A, the OR for large cancer vs. small cancer was 11.8 (95%CI 2.7 to 51.8) after multiple adjustments including overall density. For interval cancer vs. screen-detected cancer, the OR was 1.7 (95%CI 0.4 to 8.1) with *p* = 0.218 for the linear trend.

**Table 3:**
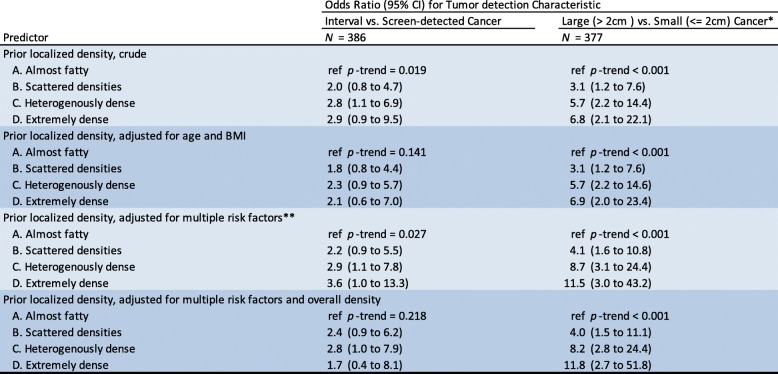
Localized density as predictor of interval cancer and large cancer. Odds ratios estimated by logistic regression modeling

Localized density based on a visual assessment of the site in the prior negative screening corresponding to subsequent tumor location, categorized according to the definitions in the American College of Radiology Breast Imaging Reporting and Data System (BI-RADS) lexicon, fifth edition. Overall density was categorized into high and low based on the cut-point (18%) representing the transition from BI-RADS category B to C

*Tumor size was based on post-operative specimen pathology assessment; missing for 8 cases

**Adjusted for age, menopausal status, body mass index, age at menarche, alcohol use, parity, use of hormone replacement therapy, use of oral contraceptives, family history, and benign breast disease

In Table 4, the regression models with interval cancer compared to screen-detected cancer have been stratified to analyze first-year (*n* = 131) and second-year (*n* = 115) interval cancers separately. Localized density shows a significant linear trend for the association with first-year interval cancers (*p* = 0.027; fully adjusted) but not for second-year interval cancers (*p* = 0.523; fully adjusted).

**Table 4:**
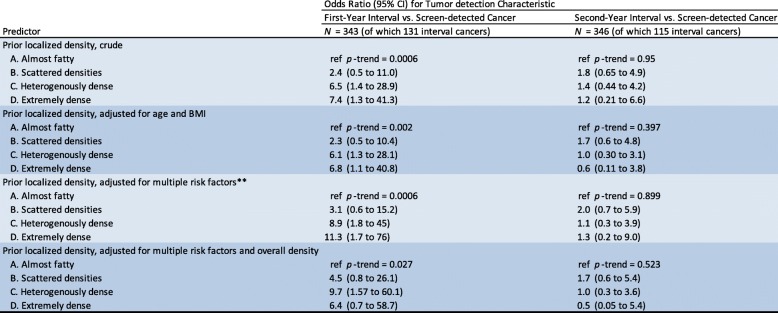
Localized density as predictor of first-year and second-year interval cancer compared to screen-detected cancer. Odds ratios estimated by logistic regression modeling

Localized density based on a visual assessment of the site in the prior negative screening corresponding to subsequent tumor location, categorized according to the American College of Radiology Breast Imaging Reporting and Data System (BI-RADS) lexicon, fifth edition. Overall density was categorized into high and low based on the cut-point (18%) representing the transition from BI-RADS category B to C

*Tumor size was based on post-operative specimen pathology assessment; missing for 8 cases

**Adjusted for age, menopausal status, body mass index, age at menarche, alcohol use, parity, use of hormone replacement therapy, use of oral contraceptives, family history, and benign breast disease

As a sensitivity analysis, we examined the effect on the association with interval cancer depending on the presence or absence of pre-existing tumor signs in the prior mammogram. The results are presented in Additional file 2: Table S2, where we dichotomized localized density into categories D and C vs. B and A in order to handle statistical power issues. The OR point estimates for interval cancer vs. screen-detected cancer showed only small differences when comparing women with tumor sign present compared to not present. The effect of localized density on the outcome of having a large, rather than small, cancer was higher among women with no prior tumor signs.

## Discussion

In this study, we have confirmed the strong association between overall density and the interval cancer—compared to healthy women as well as compared to screen-detected cancer. In the localized density-assessed subset of 386 women, we found that localized density at the site of the subsequent cancer was often different from the overall density. Importantly, we showed that localized density was independently associated with having a large cancer overall and for being diagnosed with interval cancer among first-year interval cancers.

The correlation between overall and localized density was moderate (Fig. 2). The probability of having discordant localized density and overall density was 51% overall. For women with least dense breasts, a majority of cancers arose in a localized density of higher category. This result agrees with a prior study showing that some women with low density have regions of high density that may mask cancers [27]. For women with category B below average overall density, 21% had a higher localized density. For women with category C above average overall density, 57% had a lower localized density. If screening strategies are assigned on the basis of overall density alone, these women would be at risk of being either “under-screened” or “over-screened.”

Cancer size is known to be a strong predictor of breast cancer survival [17].We confirmed findings in prior studies that large cancers are more often diagnosed in women with high overall density [28, 29]. Our most striking finding was a very strong association between localized density and large cancer, independent of the overall density (Table 4). Possibly, localized density could mask a cancer from early mammographic detection, giving it time to become larger until clinical detection or until the next mammographic screening. It is conceivable that in some cases the localized density in the prior mammogram did not mask, but actually represented, undiagnosed cancer. Limiting the analysis to the women that did not show any cancer sign at retrospective review did not markedly change the estimated effect of localized density on tumor size (Additional file 2: Table S2).

Our findings that overall density was associated with interval cancer compared to remaining healthy, and compared to being screen-detected, confirm results in prior studies [2–5, 30]. Interval cancer can emerge due to masking by overlapping density at the prior screening and due to fast growth rate [12–14]. The association between localized density and mode of detection, i.e., interval cancer compared to screen-detected cancer, was only significant for first-year interval cancers (Table 4). This finding is an extension of the results in a prior study showing that overall density has a stronger association to first-year compared to second-year interval cancers [31]. It is reasonable to believe that the association between localized density and interval cancer is mainly related to non-visible masked cancers that existed already at the prior screening rather than to true fast-growing interval cancers.

In addition to localized masking, density has been shown to be a biomarker for cancer-promoting tissue characteristics [31–34]. Speculatively, overall density might be a more accurate biomarker than localized density for biochemical exposures that affect the entire breast gland. One supporting histological study showed that changes in serum hormone levels were driving small changes in the entire breast tissue rather than larger focal changes [35].

Strengths of our study include that it was based on a prospective cohort, that the Swedish personal numbers could be used for complete follow-up, that we had information on an extensive range of potential confounders, and that we employed a novel method of assessing the localized density. A weakness of our study is that the visual localized density assessments are subjective and may vary between radiologists. A limitation of our study was that we were not able to retrieve the mammograms of 471 cases. This was due to having an examination date prior to the consent date, and not due to any criteria applied to the outcome or the density predictor. Therefore, we expect the major effect to be a loss of statistical power. A potential bias in our study is that there is a possibility that a high localized density is actually an early manifestation of the cancer itself. We have tried to address this issue by assessing potential prior tumor signs according to the European Union guidelines for quality assurance in breast cancer screening and diagnosis. The effect of localized density on interval cancer status was similar among women with and without prior tumor signs. For cancer size, the effect of localized density was higher among women with prior tumor signs. Again, this reinforces our hypothesis that localized density exerts its effect mainly through masking.

The approach in our study was based on knowledge of the tumor location. To be useful in a screening setting, there is a need for the development of computer software that can assess localized density across the mammogram and produce summary measures on which screening decisions can be based. There is no on-going development to have the STRATUS software assess localized areas, but this study lays the ground for exploring such an approach. Computer-based approaches have been presented in recent studies in which images of simulated lesions were merged into the mammographic image after which the visibility was computer-assessed [36, 37]. The commercially available Volpara software provides local density maps that could potentially be used as a foundation for creating summary measures. Summary measures of localized density, as a predictor of delayed detection, could be combined with breast cancer risk models to identify women who might benefit the most from supplemental screening with ultrasound or MRI.

## Conclusions

Our study shows that overall mammographic density often misrepresents the localized density where breast cancer actually arises. Based on our findings, further approaches to localized assessments are likely to emerge.

## Additional files

Additional file 1: **Figure S1.** Derivation of the study population. (PNG 88 kb)

Additional file 2: **Table S1.** Tumor detection characteristics by combinations of whole-image and localized density. **Table S2.** Localized density as predictor of tumor detection characteristics - subgrouped by prior tumor signs*. Odds ratios were estimated by logistic regression modeling. (DOCX 200 kb)

## Abbreviations

BI-RADS: A four-category visual classification of mammographic density issued by the density American College of Radiology, where “A” is the least and “D” the most dense
BMI: Body mass index
CI: Confidence interval
OR: Odds ratios
