# Supplementary figures and images for: Localized mammographic density is associated with interval cancer and large breast cancer: a nested case-control study
Source: Breast Cancer Res. 2019 Jan 22;21:8. doi: 10.1186/s13058-019-1099-y (PMC6341532; doi:10.1186/s13058-019-1099-y)

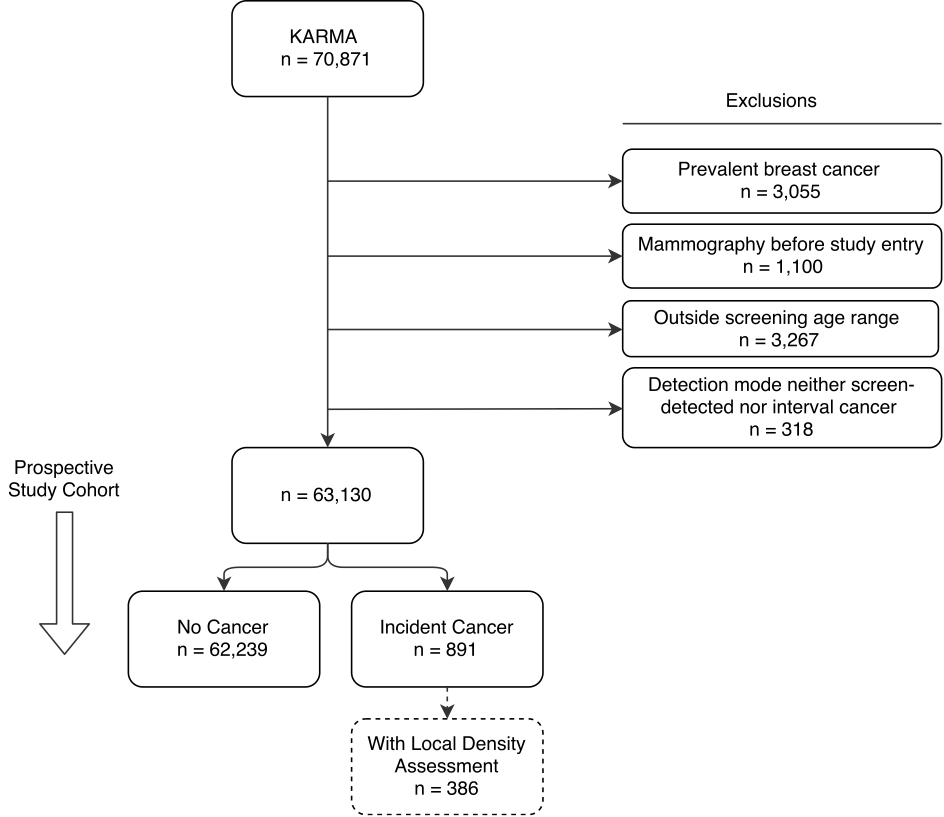

Supplement: Supplementary file 1 — Figure S1. Derivation of the study population. (PNG 88 kb) [file 13058_2019_1099_MOESM1_ESM.png]
