# Supplementary material for: Localized mammographic density is associated with interval cancer and large breast cancer: a nested case-control study
Source: Breast Cancer Res. 2019 Jan 22;21:8. doi: 10.1186/s13058-019-1099-y (PMC6341532; doi:10.1186/s13058-019-1099-y)
Supplement: Supplementary file 2 [file 13058_2019_1099_MOESM2_ESM.docx]

**Supplemental Table S1.** Tumor detection characteristics by combinations of whole-image and localized density.

| OVERALL Mammographic Density* | LOCALIZED Mammographic Density* | *N* | Interval Cancer** proportion | *N* | Large Cancer (>2cm) proportion | Tumor size, mm mean (SD) |
|---|---|---|---|---|---|---|
| A | Any localized density | 43 | 0% | 42 | 12% | 13.9 (10.5) |
| A | A | 15 | 0% | 14 | 0% | 10.5 (5.3) |
| A | B | 26 | 0% | 26 | 19% | 16.5 (15.9) |
| A | C | 2 | -- | 2 | -- | -- |
| A | D | 0 | -- | 0 | -- | -- |
| B | Any localized density | 143 | 18% | 143 | 24% | 18.4 (12.4) |
| B | A | 26 | 19% | 26 | 8% | 12.6 (8.4) |
| B | B | 86 | 20% | 86 | 28% | 18.4 (12.7) |
| B | C | 29 | 10% | 29 | 31% | 18.6 (12.6) |
| B | D | 2 | -- | 2 | -- | -- |
| C | Any localized density | 156 | 26% | 149 | 34% | 19.3 (15.3) |
| C | A | 17 | 6% | 17 | 24% | 13.7 (9.6) |
| C | B | 71 | 31% | 67 | 24% | 15.9 (8.9) |
| C | C | 59 | 29% | 57 | 46% | 22.5 (16.6) |
| C | D | 9 | 11% | 8 | 50% | 23.8 (13.7) |
| D | Any localized density | 44 | 36% | 43 | 37% | 20.1 (16.5) |
| D | A | 2 | -- | 2 | -- | -- |
| D | B | 11 | 9% | 11 | 36% | 18.0 (8.6) |
| D | C | 17 | 53% | 17 | 35% | 23.1 (14.4) |
| D | D | 14 | 36% | 13 | 46% | 28.0 (16.3) |

**) Overall density was categorized by percent density scale cutpoints (49%) into two groups reflecting the definitions by the American College of Radiology Breast Imaging Reporting and Data System (BI-RADS) lexicon, Fifth edition. Localized density based on a visual assessment of the site in the prior negative screening corresponding to subsequent tumor location.*

***) Interval breast cancer is defined as clinically detected breast cancer after a negative screening examination and before the next planned one.*

*Note: For categories with less than 5 cases, no proportions or means were calculated.*

**Supplemental Table S2.** Localized density as predictor of tumor detection characteristics - subgrouped by prior tumor signs*. Odds ratios were estimated by logistic regression modelling.

| | All cases | No tumor sign | Minimal sign + False negative |
|---|---|---|---|
| Localized density - category 'A' + 'B' | | | |
| All cases | 254 | 178 | 76 |
| Interval Cancer | 93 | 36 | 11 |
| Screen-detected Cancer | 161 | 142 | 65 |
| Large Cancer | 102 | 35 | 20 |
| Small Cancer | 147 | 138 | 56 |
| Localized density - category 'C' + 'D' | | | |
| All cases | 132 | 69 | 36 |
| Interval Cancer | 36 | 23 | 12 |
| Screen-detected Cancer | 96 | 56 | 39 |
| Large Cancer | 51 | 32 | 19 |
| Small Cancer | 77 | 46 | 30 |
| | OR (95%CI) | OR (95%CI) | OR (95%CI) |
| Interval cancer vs. Screen-detected cancer | | | |
| OR density (D and C) : (B and A) | 1.6 (1.0 to 2.7) | 1.6 (0.9 to 3.0) | 1.8 (0.7 to 4.5) |
| Large cancer vs. Small cancer | | | |
| OR density (D and C) : (B and A) | 2.4 (1.5 to 3.8) | 2.7 (1.5 to 4.9) | 1.8 (0.8 to 3.8) |

The density categories were defined according to BI-RADS into 'A': Almost fatty, 'B': Scattered densities, 'C': Heterogenously dense 'D': Extremely dense

*) Tumor signs in the prior negative screening mammogram were classified in accordance with the European Union guidelines for quality assurance in breast cancer screening and diagnosis
